# Effect of regulating airway pressure on intrathoracic pressure and vital organ perfusion pressure during cardiopulmonary resuscitation: a non-randomized interventional cross-over study

**DOI:** 10.1186/s13049-015-0164-5

**Published:** 2015-10-28

**Authors:** Younghoon Kwon, Guillaume Debaty, Laura Puertas, Anja Metzger, Jennifer Rees, Scott McKnite, Demetris Yannopoulos, Keith Lurie

**Affiliations:** Department of Medicine-Cardiovascular Division, University of Minnesota, Minneapolis, MN USA; Department of Medicine-Cardiovascular Division, University of Virginia, Charlottesville, VA USA; UJF-Grenoble 1/CNRS/CHU de Grenoble/TIMC-IMAG UMR 5525, Grenoble, France; Department of Emergency Medicine, University of Minnesota, CMinneapolis, MN USA

**Keywords:** Airway pressure, Cardiopulmonary resuscitation, Impedance threshold device, Intrapleural pressure, Intrathoracic pressure regulation

## Abstract

**Background:**

The objective of this investigation was to evaluate changes in intrathoracic pressure (Ppl), airway pressure (Paw) and vital organ perfusion pressures during standard and intrathoracic pressure regulation (IPR)-assisted cardiopulmonary resuscitation (CPR).

**Methods:**

Multiple CPR interventions were assessed, including newer ones based upon IPR, a therapy that enhances negative intrathoracic pressure after each positive pressure breath. Eight anesthetized pigs underwent 4 min of untreated ventricular fibrillation followed by 2 min each of sequential interventions: (1) conventional standard CPR (STD), (2) automated active compression decompression (ACD) CPR, (3) ACD+ an impedance threshold device (ITD) CPR or (4) ACD+ an intrathoracic pressure regulator (ITPR) CPR, the latter two representing IPR-based CPR therapies. Intrapleural (Ppl), airway (Paw), right atrial, intracranial, and aortic pressures, along with carotid blood flow and end tidal CO_2_, were measured and compared during each CPR intervention.

**Results:**

The lowest mean and decompression phase Ppl were observed with IPR-based therapies [Ppl mean (mean ± SE): STD (0.8 ± 1.1 mmHg); ACD (−1.6 ± 1.6); ACD-ITD (−3.7 ± 1.5, *p* < 0.05 vs. both STD and ACD); ACD-ITPR (−7.0 ± 1.9, *p* < 0.05 vs. both STD and ACD)] [Ppl decompression (mean ± SE): STD (−6.3 ± 2.2); ACD (−13.0 ± 3.8); ACD-ITD −16.9 ± 3.6, *p* < 0.05 vs. both STD and ACD); ACD-ITPR −18.7 ± 3.5, *p* < 0.05 vs. both STD and ACD)]. Interventions with the lower mean or decompression phase Ppl also demonstrated lower Paw and were associated with higher vital organ perfusion pressures.

**Conclusions:**

IPR-based CPR methods, specifically ACD-ITPR, yielded the most pronounced reduction in both Ppl and Paw and resulted in the most favorable augmentation of hemodynamics during CPR.

**Electronic supplementary material:**

The online version of this article (doi:10.1186/s13049-015-0164-5) contains supplementary material, which is available to authorized users.

## Background

Dynamic changes in intrathoracic pressure during cardiopulmonary resuscitation (CPR) play an important role in generating forward blood flow and thus perfusion pressure in CPR [1]. Since 1960, when closed-chest manual or standard (STD) CPR was first described, multiple new CPR methods and techniques have been developed to further enhance blood flow to the heart and brain in patients in cardiac arrest. [2–5] Despite multiple clinical advances, many of the complex underlying physiological processes responsible for maintaining and regulating circulation to these vital organs during CPR remain incompletely understood. With each chest compression, intrathoracic pressure rises and depending upon the circumstances of the arrest, including the duration and method of CPR, the heart is also physically compressed between the sternum and the spine [6]. The increase in pressure within the heart and intrathoracic blood vessels force blood forward as long as the cardiac valves remain functional [7, 8]. The subsequent chest recoil generates a small negative intrathoracic pressure that draws venous blood into the right heart which helps to refill the heart prior to the next chest compression. When the chest recoil phase starts during STD-CPR respiratory gases rush into the lungs and equilibrate the intrathoracic pressure, thereby limiting the decrease of intrathoracic pressure [9]. The degree of chest wall recoil varies from patient to patient. Based upon these mechanisms of action, STD-CPR only provides 10–30 % of normal blood flow to the brain and heart. This low rate of cardio-cerebral perfusion likely contributes to the persistently low neurologically-sound survival rates typically reported with the STD-CPR [10].

Building upon the physiological underpinning of STD-CPR, newer methods have been developed to enhance the development of negative intrathoracic pressure during the recoil phase of CPR. Several novel therapies designed to regulate intrathoracic pressure have been developed and evaluated in animals and in humans. These therapies have been termed intrathoracic pressure regulation (IPR) therapies. Each one alone or in combination enhances negative intrathoracic pressure during the chest recoil phase following delivery of a positive pressure ventilation. One device, termed an impedance threshold device (ITD), impedes ambient air from passively entering the lungs during the chest recoil phase of both STD-CPR and active compression decompression (ACD) CPR [11]. Another device, termed an intrathoracic pressure regulator (ITPR), actively creates a continuous low level negative intrathoracic pressure during STD-CPR and ACD CPR following each positive pressure breath [12].

Despite the fundamental importance of changes in intrathoracic pressure during CPR, intrathoracic pressure has generally been indirectly assessed by measuring changes in airway pressure (Paw) as a surrogate [12, 13]. True intrathoracic pressure can be measured in the intrapleural space (Ppl) and indirectly in the esophagus [13, 14]. In an effort to better understand the mechanisms of pressure transduction during the chest compression and decompression phases of conventional CPR and some newer IPR methods of CPR, we directly measured and examined the relationship between overall changes Ppl and Paw while performing these CPR methods in a pig model of cardiac arrest. We tested the hypothesis that overall intrathoracic pressure change as measured by Ppl would be reflected in Paw during CPR techniques including IPR therapy. We further hypothesized that the greatest reduction in Ppl and Paw during the decompression phase of CPR created by using IPR devices would result in the highest calculated coronary (CPP) and cerebral perfusion pressures (CePP).

## Methods

This study was approved by the Institutional Animal Care Committee of the Minneapolis Medical Research Foundation of Hennepin County Medical Center. All animal care was compliant with the National Research Council’s 1996 Guidelines for the Care and Use of Laboratory Animals. A certified and licensed veterinarian assured the protocols were performed in accordance with the National Research Council’s Guidelines.

### Animal instrumentation

The surgical preparation, anesthesia, data monitoring, and recording procedures used in this study have been previously described [15]. Eight female domestic farm pigs (34 ± 2 kg) were fasted overnight. Each animal received an intramuscular injection 10 ml (100 mg/ ml) of ketamine HCl (Ketaset, Fort Dodge Animal Health, Fort Dodge, Iowa) for initial sedation. Anesthesia induction with 5 % isoflurane was then delivered through a mask. The animal was subsequently intubated while spontaneously breathing with a 7.0 French endotracheal tube. Isoflurane was then continuously administered at a dose of 0.8–1.2 % for maintenance of general anesthesia. An ear vein was cannulated for venous access. Animals were ventilated with a tidal volume of 10 mL/kg at a rate of 12 (Narcomed, Telford, Pennsylvania) through the preparatory period. After creating a burr hole, an intracranial bolt was positioned at the right parietal aspect of the cranium and a micromanometer-tipped catheter (Mikro-Tip® Transducer, Millar Instruments, Inc. Houston, TX) was inserted into the parietal lobe to measure intracranial pressure (ICP). The left femoral artery and right external jugular veins were cannulated using a modified Seldinger percutaneous technique. Central aortic blood pressure was recorded continuously with a Mikro-Tip® catheter. The catheter was placed in the chest cavity at the level of the origin of the descending thoracic aorta. Using jugular access, a Mikro-Tip® catheter was inserted into the RA for recording Pra. Paw was measured with a pneumatic pressure transducer connected to the endotracheal tube. Under fluoroscopic guidance using a modified Seldinger percutaneous technique, a Mikro-Tip® catheter was placed into the right anterior aspect of the intrapleural space to measure Ppl. Excellent agreement between Ppl and Paw was demonstrated during an airway occlusion test (spontaneous breathing efforts against closed airway). Proper catheter placements were confirmed in all animals post mortem. Ppl, Paw, Pra, aortic and intracranial (ICP) pressures were recorded simultaneously using a digital data acquisition system (BIOPAC MP 150, BIOPAC Systems, Inc., CA, USA). The left common carotid artery was surgically exposed and a Doppler flow probe (Transonic, 400-Series Multi-channel, transonic Systems Inc. Ithaca, NY) was placed around it to quantify carotid blood flow (CBF). End tidal CO2 (ETCO_2_) was continuously measured with a respiratory monitor (CO2SMO Plus, Novametrix Medical Systems, Wallingford, Connecticut).

### Study protocol and measurements

**Fig. 1:**
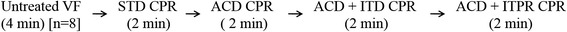
Study protocol. VF, ventricular fibrillation; STD, standard; CPR, cardiopulmonary resuscitation; ACD, active compression decompression; ITD, impedance threshold device; ITPR, intrathoracic pressure regulator

Following 4 min of untreated ventricular fibrillation, CPR was performed in the following fixed sequence for 2 min epochs for each of the following interventions: (1) Standard CPR (STD), (2) Active compression decompression (ACD) CPR alone, (3) ACD + inspiratory impedance threshold device (ITD) CPR, and (4) ACD + intrathoracic pressure regulator (ITPR) CPR (Fig. 1). An ITD with 16 cm H_2_O inspiratory resistance (ResQPOD-16™, Advanced Circulatory Systems, Roseville, MN) was used in these studies. The ITPR device used in this evaluation, the CirQLator™ (Advanced Circulatory Systems, Roseville, MN), required a continuous vacuum source and a means to provide intermittent positive pressure ventilation. This device has been described previously [12, 16, 17]. It is inserted between the endotracheal tube and the ventilator. STD-CPR and ACD-CPR were performed with a pneumatically driven automatic piston device (Pneumatic Compression Controller, Ambu International, Glostrup, Denmark) as previously described [11]. During standard CPR, uninterrupted chest compressions were performed at a rate of 100 compressions/min, with a 50 % duty cycle and a compression depth of 25 % of the anteroposterior chest diameter. After each compression the chest wall was allowed to fully recoil passively. ACD-CPR was performed with a 9.0-cm silicon suction cup. Compression and decompression excursions were continuously monitored and adjusted with the control module, during the experiment. Uninterrupted chest compressions were performed during ACD CPR at a rate of 100 compressions/min, with a 50 % duty cycle and a compression depth of 25 % of the antero-posterior chest diameter. After each compression the chest wall was actively pulled upward to produce a sternal displacement of 10 % greater than the resting anterior-posterior diameter.

Data from two consecutive compression-decompression cycles were measured every 30 s throughout each intervention. Each data measurement included mean Ppl, Paw, Pra, aortic and intracranial pressure (ICP) over an entire duty cycle and compression (peak point) and decompression (nadir point) Ppl, Pra, Paw and aortic pressure. Transmural Pra (Pra tm) was calculated as Pra minus Ppl. CPP and CePP were calculated as diastolic aortic pressure minus diastolic RA pressure and mean aortic pressure minus mean ICP, respectively. ICP (and CePP) was measured in 6 animals. Mean CBF and ETCO_2_ for each intervention were also measured. Animals were euthanized with intravenous KCL at the conclusion of experiments.

### Statistical analysis

Average values of 8 measurements per intervention were derived and used for data analysis. Data are presented as mean ± SEM. All the aforementioned measurements during ACD, ACD + ITD and ACD + ITPR were compared to each other using repeated measures of ANOVA with a Tukey post-test. Using 32 data points (8 measurements per each of 4 CPR interventions) we derived Pearson Product-moment correlation 1) between Ppl and Paw 2) between decompression Ppl and Paw, and calculated vital organ perfusion pressures (CPP and CePP). P-value less than 0.05 was considered statistically significant.

## Results

Key hemodynamic measurements are shown in Table 1 (see Additional file 1: Table S1 for baseline [before arrest] hemodynamic data). Overall, circulation as measured by CPP, CePP, CBF, and ETCO_2_ improved with the use of ACD-ITD or ACD-ITPR.

**Table 1:** Summary of hemodynamic data

	STD	ACD	ACD-ITD	ACD-ITPR
SBP	59.4 ± 7.7	63.1 ± 7.9^*^	71.3 ± 9.8^*,**^	75.4 ± 11.6^*,**^
DBP	21.7 ± 3.6	16.4 ± 3.0	21.5 ± 4.6	23.8 ± 5.4^**^
Pra max	58.2 ± 7.6	68.1 ± 10.5^*^	70.4 ± 9.6^*^	74.3 ± 9.8^*^
Pra min	1.8 ± 1.6	−0.5 ± 1.7	−1.4 ± 1.6*	−2.4 ± 1.4^*^
Pra mean	23.3 ± 2.9	21.8 ± 3.8	22.5 ± 3.3	23.7 ± 3.6
ICP	20.9 ± 1.1	19.0 ± 1.5	19.7 ± 1.7	19.9 ± 2.1
CePP	13.0 ± 3.1	13.2 ± 3.0	17.1 ± 4.1	19.0 ± 5.3^*,**^
CPP	12.9 ± 3.0	11.6 ± 1.7	16.7 ± 2.9^**^	18.1 ± 3.6^*,**^
CBF	37.7 ± 6.4	48.8 ± 8.7^*^	53.3 ± 9.0^*^	57.4 ± 10.5^*^
ETCO_2_	21.5 ± 2.2	23.7 ± 3.1	30.3 ± 1.9^*,**^	30.3 ± 3.5^*,**^

Values are shown as mean ± SEM. All pressures are in mm Hg and all flows in mL/min. Data are derived from 8 animals except for ICP, CePP and ETCO_2_ (*n* = 6)

*STD* standard, *ACD* active compression decompression, *ITD* impedance threshold device, *ITPR* intrathoracic pressure regulator, *SBP* and *DBP* systolic and diastolic aortic blood pressure, *Pra* right atrial pressure, *ICP* intracranial pressure, *CePP* cerebral perfusion pressure, *CPP* coronary perfusion pressure, *CBF* carotid blood flow, *ETCO*_*2*_ end-tidal CO_2_; max, maximum,;min, minimum

*denotes *p* < 0.05 for STD vs. (ACD, ACD ITD, ACD ITPR)

**denotes *p* < 0.05 for ACD vs. (ACD ITD, ACD ITPR)

### Intrapleural (Ppl) and airway pressure (Paw) during the four CPR interventions

**Fig. 2:**
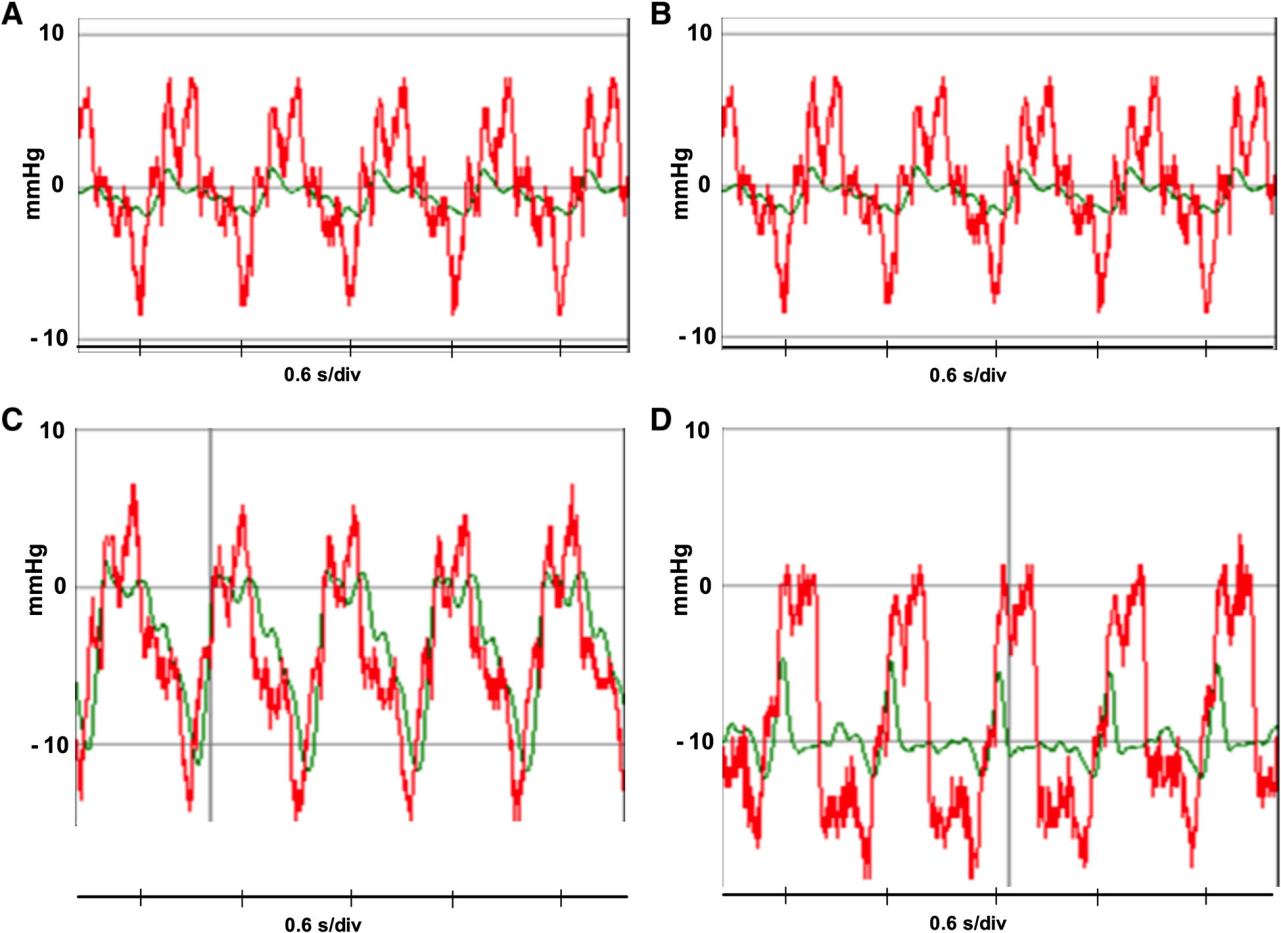
Representative change in Ppl (Intrapleural pressure) (red) and Paw (airway pressure) (green) during CPR. **a** Standard (STD); **b** Active compression decompression (ACD); **c** ACD-Impedance Threshold Device (ITD); **d** ACD-Intrathoracic Pressure Regulator (ITPR)

The relationship between Ppl and Paw varied, depending upon the method of CPR. In general there was a high degree of concordant change within a duty cycle between these two pressure measurements. A representative example of simultaneously recorded Ppl and Paw during the 4 CPR interventions is shown in Fig. 2.

Ppl had a larger range in peak-to-trough values (∆) during each compression and decompression duty cycle compared with Paw. The change in Ppl varied across the CPR interventions (Table 2). ∆ Ppl during STD CPR was smaller compared with all other CPR interventions (Table 2). There were no differences in ∆ Ppl among the three ACD CPR interventions.

**Table 2:** Intrapleural and airway pressure during sequential CPR interventions

	STD	ACD	ACD-ITD	ACD-ITPR
Ppl mean	0.8 ± 1.1	−1.6 ± 1.6	−3.7 ± 1.5^*,**^	−7.0 ± 1.9^*,**,***^
Ppl max	6.8 ± 0.9	9.4 ± 1.3	8.4 ± 1.2	5.9 ± 1.8^**^
Ppl min	−6.3 ± 2.2	−13.0 ± 3.8^*^	−16.9 ± 3.6^*,**^	−18.7 ± 3.5^*,**^
Ppl delta	13.1 ± 2.2	22.5 ± 3.5^*^	26.2 ± 4^*^	24.6 ± 3.3^*^
Paw mean	0.3 ± 0.3	0.3 ± 0.3	−2.0 ± 0.6^*,**^	−8.4 ± 1.0^*,**,***^
Paw max	1.4 ± 0.3	2.2 ± 0.4	3.3 ± 1.1	−5.1 ± 1.4^*,**,***^
Paw min	−0.9 ± 0.3	−1.4 ± 0.3	−9.4 ± 0.8^*,**^	−11.9 ± 0.8^*,**,***^
Paw delta	2.3 ± 0.3	3.6 ± 0.4	12.6 ± 1.1^*,**^	6.4 ± 1.4^*,**,***^

Values are shown as mean ± SEM (mm Hg). All pressures are in mm Hg. Data are derived from 8 animals

*CPR* cardiopulmonary resuscitation, *STD* standard, *ACD* active compression decompression, *ITD* impedance threshold device, *ITPR* intrathoracic pressure regulator, *Ppl* intrapleural pressure, *Paw* Airway pressure; max, maximum (compression); min, minimum (decompression)

*denotes *p* < 0.05 for STD vs. (ACD, ACD ITD, ACD ITPR)

**denotes *p* < 0.05 for ACD vs. (ACD ITD, ACD ITPR)

***denotes *p* < 0.05 for ACD-ITD vs. ACD-ITPR

The ∆ Paw was minimal during STD CPR and ACD CPR (Table 2). By contrast, compared to either STD CPR or ACD CPR, ∆ Paw was more prominent during the ACD-ITD and ACD-ITPR interventions. The largest ∆ Paw was observed during ACD-ITD intervention (Table 2), with Paw change closely tracking the Ppl with each compression and decompression (Table 2, Fig. 2c).

**Fig. 3:**
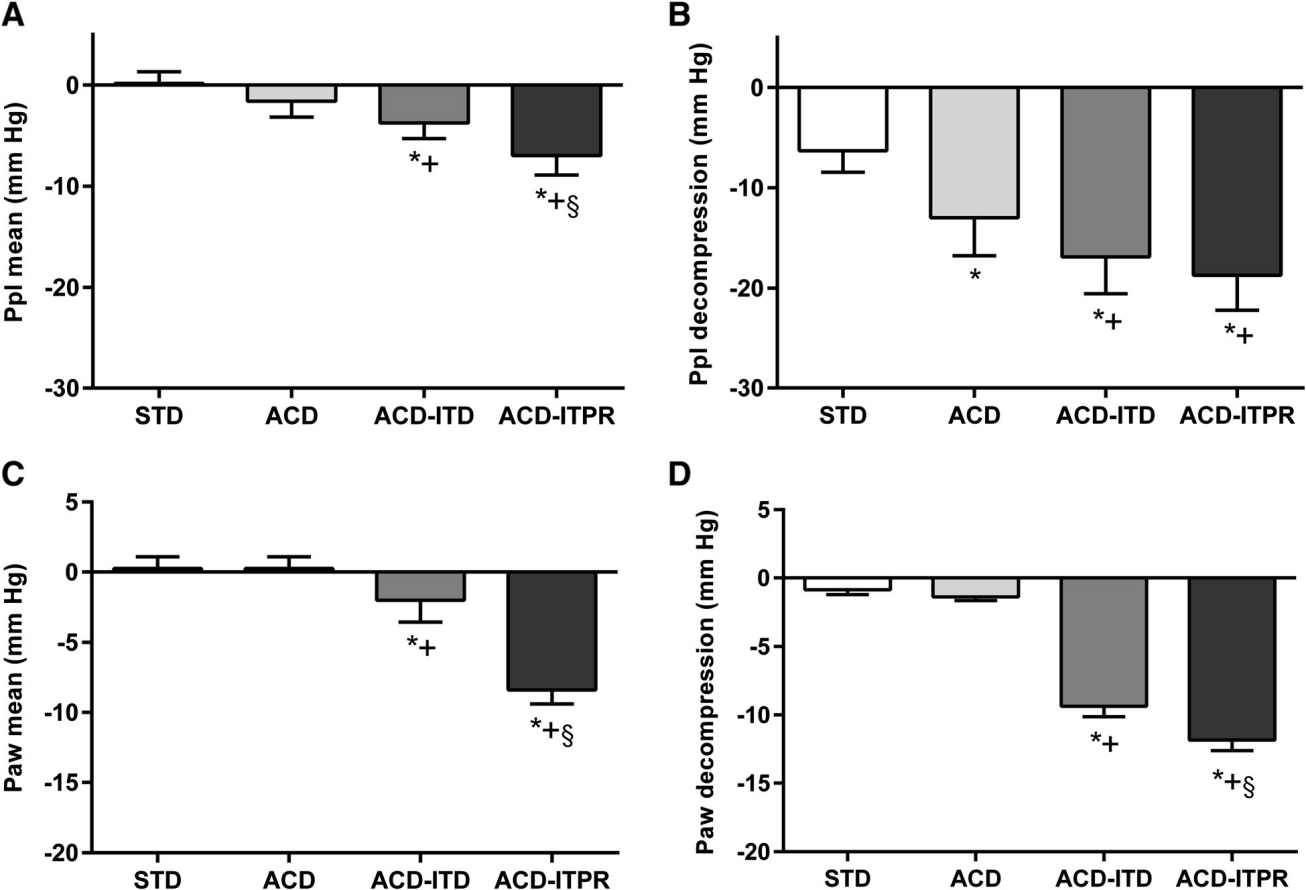
Ppl (Intrapleural pressure) and Paw (airway pressure) with each intervention. **a**. Ppl mean; **b**. Ppl decompression; **c**. Paw mean; **d**. Paw decompression. Error bar represents SEM. * denotes *p* < 0.05 for STD vs. (ACD, ACD ITD, ACD ITPR). + denotes *p* < 0.05 for ACD vs. (ACD ITD, ACD ITPR). § denotes *p* < 0.05 for ACD-ITD vs. ACD-ITPR. P values for linear trend were all < 0.05. STD, standard; ACD, active compression decompression, ITD, impedance threshold device; ITPR, intrathoracic pressure regulator

Figure 3 shows mean Ppl and Paw during the series of 4 CPR interventions. ACD-ITPR CPR resulted in the most negative mean and decompression Ppl. Compared with STD or ACD CPR, mean Ppls of ACD-ITD and ACD-ITPR CPR were more negative. In addition the decompression Ppl of ACD CPR was more negative than that of STD CPR. In turn, the decompression Ppls of ACD-ITD and ACD-ITPR CPR were more negative than that of ACD CPR (Table 2). Similar findings were observed with the mean and decompression Paw. Mean Paws of STD and ACD-CPR were close to a reference pressure point, 0 mmHg (0.3 ± 0.3 and 0.3 ± 0.3, respectively). Both mean and decompression Paws of the ACD-ITD (−2.0 ± 0.6; −9.4 ± 0.8 mmHg) and the ACD-ITPR CPRs (−8.4 ± 1.0; −11.9 ± 0.8 mmHg) were more negative compared with that of either STD or ACD CPR (Table 2) (see Additional file 1: Table S2 for baseline [before arrest] Ppl and Paw).

### Right atrial pressure (Pra) during the four CPR interventions

**Fig. 4:**
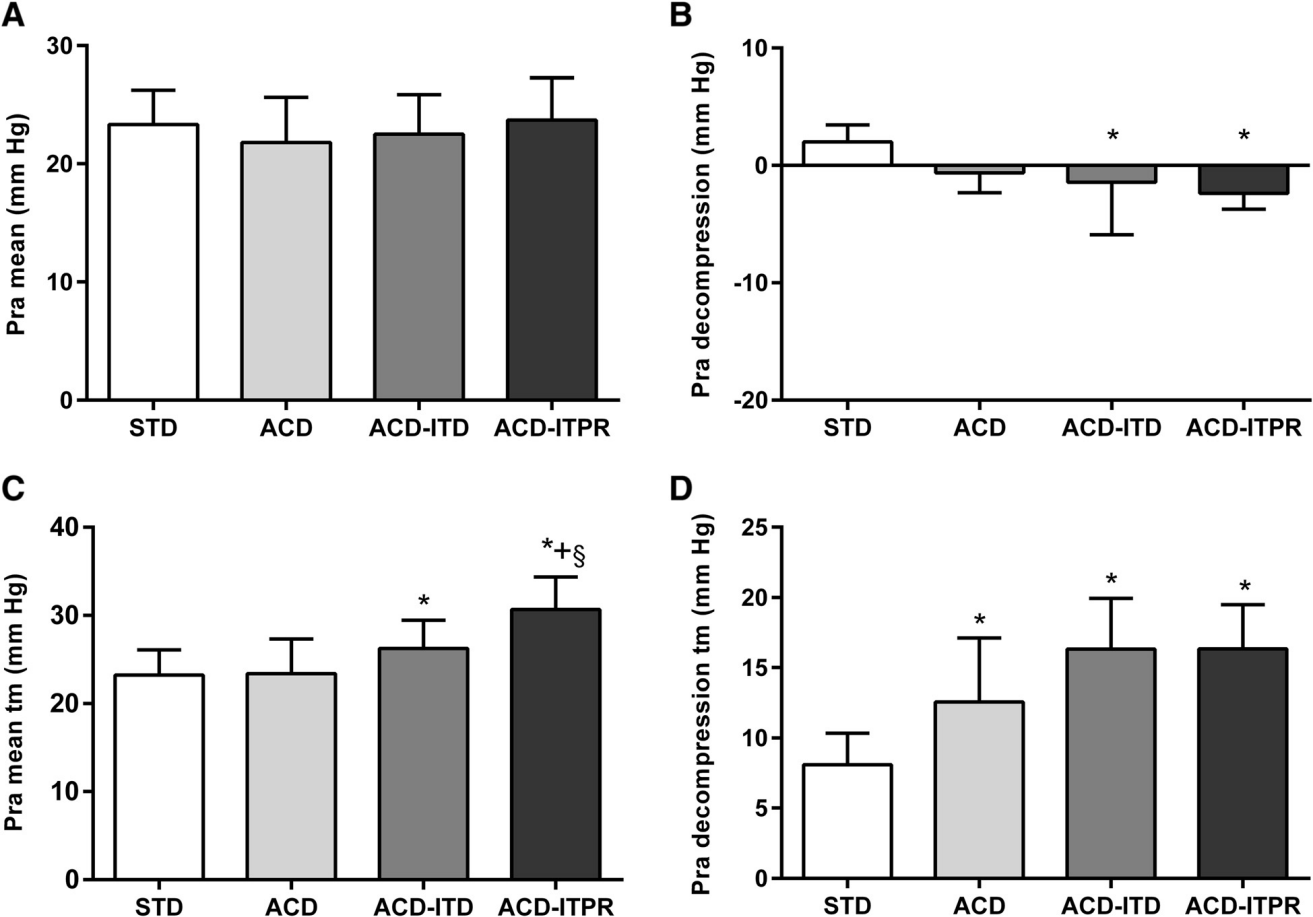
Pra (Right atrial pressure) with each intervention. **a**. Pra mean; **b**. Pra decompression; **c**. Transmural Pra mean (Pra mean tm); **d**. Transmural Pra decompression (Pra decompression tm). Error bar represents SEM. * denotes *p* < 0.05 for STD vs. (ACD, ACD ITD, ACD ITPR). + denotes *p* < 0.05 for ACD vs. (ACD ITD, ACD ITPR). § denotes *p* < 0.05 for ACD-ITD vs. ACD-ITPR.. P values for linear trend were all < 0.05 except in A.STD, standard; ACD, active compression decompression, ITD, impedance threshold device; ITPR, intrathoracic pressure regulator

Absolute values of mean Pra were considerably higher (STD: 23.3 ± 2.9; ACD: 21.8 ± 3.8; ACD-ITD: 22.5 ± 3.3; ACD-ITPR; 23.7 ± 3.6 mmHg) than mean Ppl and Paw (Table 1, Fig. 4a). However, unlike mean Ppl and Paw, there were no significant observed differences across the 4 CPR interventions. In addition, absolute decompression Pra was less negative than Ppl or Paw. However, decompression Pra was lower during ACD-ITD (−1.4 ± 1.6 mmHg) and ACD-ITPR CPR (−2.4 ± 1.4 mmHg) compared with either STD or ACD CPR (Table 1, Fig. 4b). Mean transmural (tm) Pra (Pra mean-Ppl mean) was higher during ACD-ITD (26.2 ± 3.2 mmHg) and ACD-ITPR CPR (30.7 ± 3.7 mmHg) as compared with either STD-CPR (23.2 ± 2.9 mmHg) and ACD CPR alone (23.4 ± 3.9 mmHg) (Fig. 4c). In contrast to Pra decompression, Pra decompression tm (Pra decompression-Ppl decompression) demonstrated an opposite pattern with a significant increase during ACD (12.6 ± 4.5 mmHg), ACD-ITD (16.3 ± 3.6 mmHg) and the ACD-ITPR CPR (16.3 ± 3.1 mmHg) compared with STD-CPR (8.1 ± 2.3 mmHg) (Fig. 4d).

### Vital organ perfusion pressures; coronary perfusion pressure (CPP) and cerebral perfusion pressure (CePP) during the four CPR interventions

**Fig. 5:**
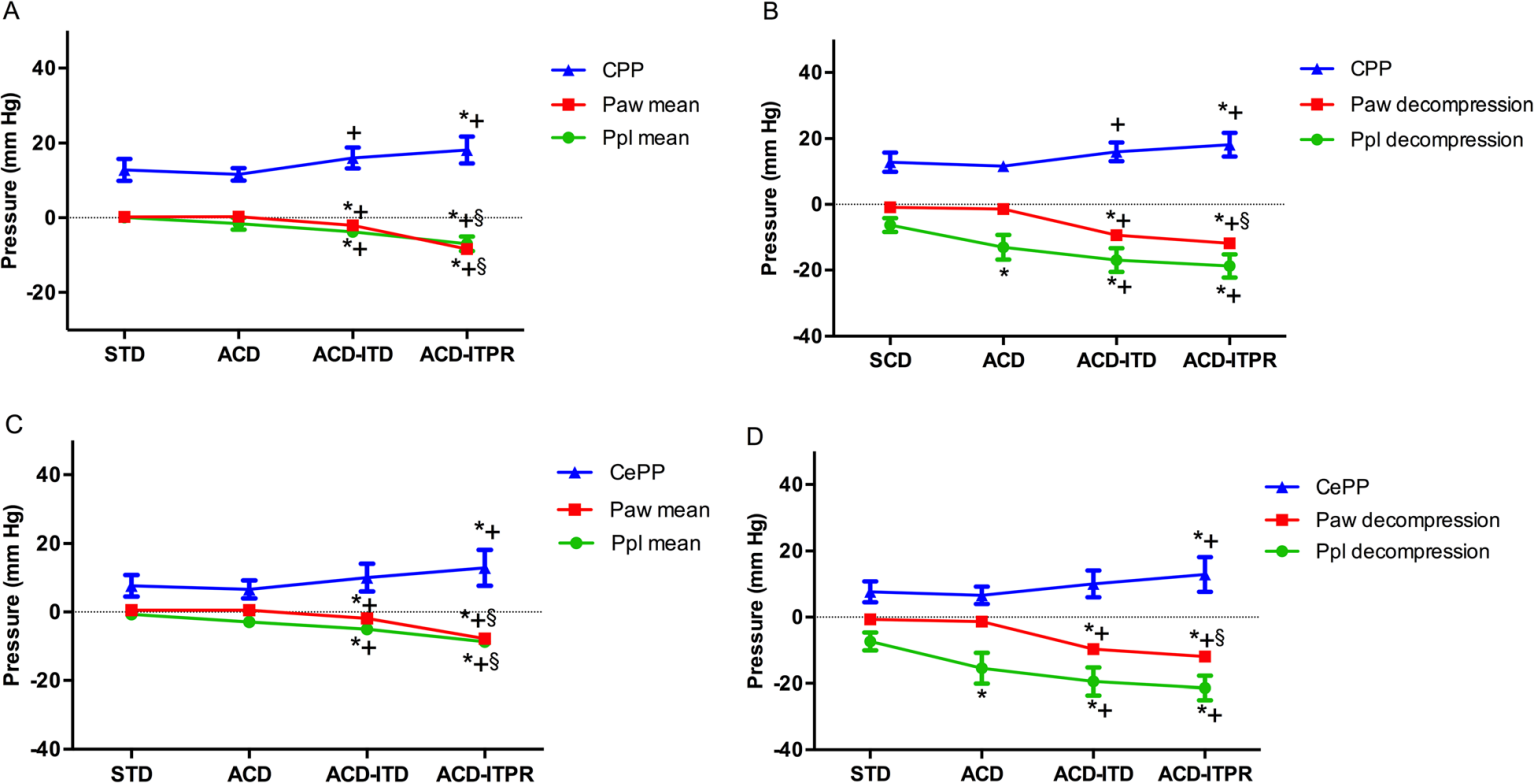
Ppl (Intrapleural pressure), Paw (Airway pressure) and vital organ perfusion pressure for each sequential intervention. **a**. Ppl mean, Paw mean and CPP (coronary perfusion pressure); **b**. Ppl decompression, Paw decompression and CPP; **c**. Ppl mean, Paw mean and CePP (cerebral perfusion pressure); **d**. Ppl decompression, Paw decompression and CePP. Error bar represents SEM. *N* = 6 for CPP. *N* = 8 for CePP. * denotes *p* < 0.05 for STD vs. (ACD, ACD ITD, ACD ITPR). + denotes *p* < 0.05 for ACD vs. (ACD ITD, ACD ITPR). § denotes *p* < 0.05 for ACD-ITD vs. ACD-ITPR.. P values for linear trend for both CPP and CePP were < 0.05. STD, standard; ACD, active compression decompression, ITD, impedance threshold device; ITPR, intrathoracic pressure regulator

Figure 5 summarizes CPP and CePP during the 4 CPR interventions in relation to Ppl and Paw. The highest CPP was observed during the ACD-ITPR CPR followed by the ACD-ITD CPR. CPP during both ACD-ITPR and ACD-ITD CPR were higher compared to either STD or ACD CPR (Table 1). ACD-ITPR CPR, but not ACD-ITD (17.1 ± 4.1 mmHg), resulted in a higher CePP compared with either STD or ACD CPR (Table 1). Higher CBF was achieved during ACD assisted interventions. Higher ETCO_2_ was achieved during ACD-ITD or ACD-ITPR interventions versus either STD or ACD CPR (Table 1).

### Correlation between Ppl and Paw, and between decompression Ppl and vital organ perfusion pressures

There was modest but significant correlation between Ppl mean vs. Paw mean (*r* = 0.42, *p* = 0.02) and Ppl decompression vs. Paw decompression (*r* = 0.36, *p* = 0.04). Ppl decompression was correlated with a trend of higher CPP (*r* = −0.33, *p* = 0.07) and significantly higher CePP (*r* = −0.69, *p* < 0.001) (Additional file 2: Figure S1).

## Discussion

During CPR cerebral and coronary blood flow is critically dependent upon changes in intrathoracic pressure. Multiple approaches have been developed in an effort to optimize circulation during CPR by modulating changes in intrathoracic pressure but little is known about the changes in absolute intrathoracic pressure during the decompression phase. In this study, we measured the relative and absolute changes in intrathoracic pressure associated with several methods of CPR. By direct measurement of pressure in the intrapleural space, a representation of true intrathoracic pressure, we tested for key assumptions which underlie the mechanism of these CPR therapies. When compared with standard CPR or ACD CPR by itself, we observed that decompression Ppl was significantly lower when ACD was combined with an ITD or an ITPR device. More negative Ppls were associated with higher vital organ perfusion pressures. The results further demonstrated modest correlation between Ppl and Paw during CPR. While Ppl has previously been measured during conventional CPR or derivative methods designed to improve flow by increasing intrathoracic pressure during chest compression, it has not been studied in the context of CPR techniques specifically designed to improve flow by decreasing intrathoracic pressure during chest decompression, such as those employing ACD in conjunction with an ITD or ITPR device [7, 18, 19]. These new CPR techniques have been shown to significantly improve hemodynamics during CPR and enhance survival with a good neurologic outcome [5, 16, 17, 20–22]. In an effort to explain the improved circulation with these therapies, Paw has been mainly used as a surrogate for negative intrathoracic pressure [21]. While a positive relationship between Paw and Ppl is generally expected during positive pressure ventilation, the exact relation in the setting of CPR, particularly when the Paw is actively regulated either via ITD or negative airway pressure regulator like the ITPR, can be more complex. Although the exact mechanism of benefit of these therapies is not entirely clear, increased venous return and a reduction in intracranial pressure as a result of a more negative intrathoracic pressure constitute an important hypothesis [11, 12, 23]. Consequently, understanding the Ppl change is essential to optimize these therapies.

As demonstrated in the study results, changes in Paw and Ppl were negligible during both STD-CPR and during ACD-CPR. This is understandable as changes in intrathoracic pressure resulting from chest compressions and decompressions will be minimally reflected in the proximal airway of a patent and unobstructed airway. Subtle increases in Paw noted with ACD-CPR likely reflect an increased air flow associated with this device [24]. In contrast, during impedance of air flow such as with ITD-assisted CPR, the Paw is no longer merely a reflection of passive air flow but represents an active pressure regulation process. This explains the similar changes of Paw and Ppl during ACD-ITD CPR. In the case of ITPR, application of continuous negative tracheal pressure (negative airway pressure ventilation) results in a reduced lung volume between positive ventilations [12]. Because of this, Ppl is significantly reduced, essentially creating a negative intrathoracic atmosphere throughout the duty cycles between positive ventilations.

### ACD

ACD-CPR employs a suction device that causes active decompression of the chest after each compression. We observed greater excursions of Ppl with ACD-CPR as compared with STD-CPR, similar to previous report. [24] While compression Ppl was similar to that seen with STD-CPR, decompression Ppl was significantly lower with ACD-CPR compared with STD-CPR, leading to overall trend of lower mean Ppl. This finding is consistent with the concept that ACD reduces intrathoracic pressure by “active decompression” during recoil phase of the CPR [25]. Although the larger excursion in Ppl was reflected in Paw, the overall change in Paw was not different from STD-CPR.

### ACD-ITD

Addition of an ITD to ACD-CPR augmented the generation of negative decompression Ppl, leading to more negative mean Ppl compared to ACD alone. When placed in the airway circuit, the ITD impedes the flow of air into the lung during chest decompression. It also decreases the total amount of respiratory gas volume within the lungs during CPR since respiratory gases are pushed out of the lungs with each compression but not allowed into the lungs during the subsequent decompression as long as the ITD is in the respiratory circuit. Respiratory gases reenter the lungs during ACD-ITD only during positive pressure ventilation. Thus, the negative Paw measured during decompression is expected to be consistent with the amount of threshold impedance below which air flow will be prohibited. We observed that the Ppl and Paw were tightly correlated with the absolute amount of Ppl change during decompression exceeding that of Paw. This finding for the first time verifies the assumption that more negative intrathoracic pressure is indeed generated during the decompression phase by preventing gas inflow back into lung with an ITD.

Improvement in vital organ perfusion pressures by ITD shown in this study can be explained by the augmented negative intrathoracic pressure that we observed [5, 26–28]. Although lung volumes were not directly measured in our study, inhibition of airflow back to chest would undoubtedly reduce the volume of respiratory gases in the lungs during the CPR decompression phase. The resultant expansion of potential intrapleural space would then create negative intrathoracic pressure which in turn enhances venous return.

### ACD-ITPR

Unlike the iron lung or other negative thoracic chamber devices which generate negative intrathoracic pressure by outward expansion of chest wall, the ITPR device does so inwardly by actively withdrawing respiratory gases after each positive pressure ventilation, thereby reducing lung volume which in turn expands the potential intrapleural space [12, 21]. Constant negative Paw applied between positive ventilations resulted in a marked negative shift of Ppl. As a result, the lowest Ppl was achieved with ACD plus ITPR CPR compared with the other interventions. In addition, the highest vital organ perfusion pressures were observed during this intervention. In addition to enhanced venous return by maintaining negative intrathoracic pressure in similar manner to ITD, there may be additional unexplained mechanisms conferring hemodynamic benefit that warrant further studies. Simmons et al., in their efforts to investigate the relationship between the lung volume and pulmonary vascular resistance (PVR) under normal physiological conditions and not during CPR, demonstrated higher PVR when lung volume was reduced by applying negative expiratory pressure. Intriguingly, under this condition, both the transpulmonary pressure gradient, (pulmonary artery pressure-left atrial pressure) and to a lesser degree cardiac output, increased [29]. Taken together, the current findings and the work of Simmons et al. suggest that reduced lung volume generated by applying negative expiratory airway pressure may result in an overall increase of circulating volume by 1) increasing venous return and 2) increasing pulmonary circulation (facilitating blood flow from pulmonary vasculature to the left side of the heart), despite the rise in PVR [15].

### Right atrial pressure

Because the right atrium is a highly compliant structure, Pra is under the direct influence of the surrounding intrathoracic pressure (Ppl) and thus could be considered as a surrogate measurement for intrathoracic pressure during CPR. In addition, Pra is affected by venous blood volume returning to right atrium [30, 31]. In this context, transmural Pra, which removes the effect of Ppl, would reflect the effective circulating intravascular volume in a given right atrial compliance. In our study, transmural mean Pra was significantly higher during ACD-ITPR compared with either STD or ACD CPR, which supports enhanced venous return to the right heart as a mechanism for the effect of ITPR.

There are several limitations to the present study. First absolute Ppl represented in our study may not reflect the true Ppl due to regional variation and vertical gradient of Ppl depending on the placement. However since focus in our study was on the Ppl during the device assisted CPR relative to that during STD CPR, the results were less likely affected by this limitation. Second because of short duration of intervention, employing conventional methods of coronary and cerebral flow measurement was not technically feasible. While perfusion pressure can be used as a surrogate to blood flow, the relationship between the two is not linear due to variability in resistance. Consequently, an increase in calculated vital organ perfusion pressure with IPR-based therapies shown in our study is not equal to an increase in vital organ blow flow (i.e., the volume of transported oxygen to the cells crucial to cell survival). Related to this, metabolic factors such as gas exchange status that may affect the autonomic regulation of the blood flow (and thus distort the assumed relationship between the pressure gradient and the flow) were not measured in our study. However consistent results based on the surrogate measurement (CBF and ETCO_2_) to overall circulation suggests that the use of perfusion pressures may have been acceptable. Third, as the sequence of interventions was fixed and not randomized, there may be a time-varying confounding or preconditioning effect. The short duration of each intervention could have led to carry over effects between interventions. However, stable hemodynamic measurements seen during longer periods of series of CPR interventions in our previous studies suggest low likelihood of such effects [23, 32]. Despite these limitations, given the fact that perfusion pressure generally tends to decrease as the duration of CPR increases, our results showing the highest vital organ perfusion pressure during the last intervention (ACD-ITPR) allow us to draw reasonable conclusions. Finally, the relatively small number of animals might have resulted in lack of power to show differences in some of our statistical analyses so that caution must be used in interpreting the results that were not statistically significant.

## Conclusions

We demonstrated correlation between Paw and Ppl during the series of various CPR techniques. IPR-based CPR methods, specifically ACD-ITPR, yielded the most pronounced reduction in both Ppl and Paw and resulted in most favorable augmentation of hemodynamics during CPR. These findings suggest that lower intrathoracic pressure achieved by active airway pressure regulation constitutes a key underlying mechanism for enhanced vital organ perfusion pressure during IPR-based CPR methods.

## Additional files

Additional file 1:
**Table for “Effect of Regulating Airway Pressure on Intrathoracic Pressure and Vital Organ Perfusion Pressure during Cardiopulmonary Resuscitation: a non-randomized interventional cross-over study” by Kwon et al. Table S1**. Summary of Hemodynamic Data Including Baseline (Before Arrest). **Table S2**. Intrapleural and Airway Pressure during Baseline and Sequential CPR Interventions. (DOCX 17 kb) Additional file 2: Figure S1. Scatterplot depicting relationships between Ppl mean and Paw mean (**A**), Ppl decompression and Paw decompression (**B**), Ppl decompression and CPP (**C**) and, Ppl decompression and CePP (**D**). Ppl, Intrapleural pressure; Paw, airway pressure; CPP, coronary perfusion pressure; CePP, cerebral perfusion pressure. (DOCX 50 kb)

## Abbreviations

ACD: Active compression decompression
CePP: Cerebral perfusion pressure
CPP: Coronary perfusion pressure
IPR: Intrathoracic pressure regulation
ITD: Impedance threshold device
ITPR: Intrathoracic pressure regulator
Paw: Airway pressure
Ppl: Intrapleural pressure
Pra: Right atrial pressure
STD: Standard
