# Supplementary material for: Effect of regulating airway pressure on intrathoracic pressure and vital organ perfusion pressure during cardiopulmonary resuscitation: a non-randomized interventional cross-over study
Source: Scand J Trauma Resusc Emerg Med. 2015 Oct 28;23:83. doi: 10.1186/s13049-015-0164-5 (PMC4625943; doi:10.1186/s13049-015-0164-5)
Supplement: Additional file 1: — Table for “Effect of Regulating Airway Pressure on Intrathoracic Pressure and Vital Organ Perfusion Pressure during Cardiopulmonary Resuscitation: a non-randomized interventional cross-over study” by Kwon et al. Table S1. Summary of Hemodynamic Data Including Baseline (Before Arrest). Table S2. Intrapleural and Airway Pressure during Baseline and Sequential CPR Interventions. (DOCX 17 kb) [file 13049_2015_164_MOESM1_ESM.docx]

Supplemental Table for “Effect of Regulating Airway Pressure on Intrathoracic Pressure and Vital Organ Perfusion Pressure during Cardiopulmonary Resuscitation: a non-randomized interventional cross-over study” by Kwon et al.

Supplemental Table 1. Summary of Hemodynamic Data Including Baseline (Before Arrest)

|  | Baseline | STD | ACD | ACD-ITD | ACD-ITPR |
| --- | --- | --- | --- | --- | --- |
| SBP | 91.6±5.4 | 59.4±7.7 | 63.1±7.9* | 71.3±9.8*^┼^ | 75.4±11.6*^┼^ |
| DBP | 64.3±4.4 | 21.7±3.6 | 16.4±3.0 | 21.5±4.6 | 23.8±5.4^┼^ |
| Pra max | 5.7±1.4 | 58.2±7.6 | 68.1±10.5* | 70.4±9.6* | 74.3±9.8* |
| Pra min | 0.0±1.5 | 1.8±1.6 | -0.5±1.7 | -1.4±1.6* | -2.4±1.4* |
| Pra mean | 2.4 ± 1.5 | 21.8 ± 3.8 | 22.5 ± 3.3 | 23.7 ± 3.6 | 23.7 ± 3.6 |
| ICP | 20.3±2.5 | 20.9±1.1 | 19.0±1.5 | 19.7±1.7 | 19.9±2.1 |
| CePP | 52.3±6.3 | 13.0±3.1 | 13.2±3.0 | 17.1±4.1 | 19.0±5.3*^┼^ |
| CPP | 73.3±5.4 | 12.9±3.0 | 11.6±1.7 | 16.7±2.9^┼^ | 18.1±3.6*^┼^ |
| CBF | 185.7±37.0 | 37.7±6.4 | 48.8±8.7* | 53.3±9.0* | 57.4±10.5* |
| ETCO_2_ | 41.3±0.8 | 21.5±2.2 | 23.7±3.1 | 30.3±1.9*^┼^ | 30.3±3.5*^┼^ |
| Values are shown as mean ± SEM. All pressures are in mm Hg and all flows in mL/min. Data are derived from 8 animals except for ICP, CePP and ETCO_2_ (n=6). * denotes p< 0.05 for STD vs. (ACD, ACD ITD, ACD ITPR). ^┼^ denotes p<0.05 for ACD vs. (ACD ITD, ACD ITPR). ^§^ denotes p<0.05 for ACD-ITD vs. ACD-ITPR.  STD, standard; ACD, active compression decompression; ITD, impedance threshold device; ITPR, intrathoracic pressure regulator; SBP and DBP, systolic and diastolic aortic blood pressure; Pra, right atrial pressure; ICP, intracranial pressure; CePP, cerebral perfusion pressure; CPP, coronary perfusion pressure; CBF, carotid blood flow; ETCO_2_, end-tidal CO_2_; max, maximum; min, minimum. | | | | | |

Supplemental Table 2: Intrapleural and Airway Pressure during Baseline and Sequential CPR Interventions.

|  | Baseline | STD | ACD | ACD-ITD | ACD-ITPR |
| --- | --- | --- | --- | --- | --- |
| Ppl mean | -4.0±2.9 | 0.8±1.1 | -1.6±1.6 | -3.7±1.5*^┼^ | -7.0±1.9*^┼§^ |
| Ppl max | 0.8±1.0 | 6.8±0.9 | 9.4±1.3 | 8.4±1.2 | 5.9±1.8^┼^ |
| Ppl min | -5.7±3.0 | -6.3±2.2 | -13.0±3.8* | -16.9±3.6*^┼^ | -18.7±3.5*^┼^ |
| Ppl delta | 5.9±2.8 | 13.1±2.2 | 22.5±3.5* | 26.2±4* | 24.6±3.3* |
| Paw mean | 2.6±0.3 | 0.3±0.3 | 0.3±0.3 | -2.0±0.6*^┼^ | -8.4±1.0*^┼§^ |
| Paw max | 3.1±0.3 | 1.4±0.3 | 2.2±0.4 | 3.3±1.1 | -5.1±1.4*^┼§^ |
| Paw min | 2.1±0.3 | -0.9±0.3 | -1.4±0.3 | -9.4±0.8*^┼^ | -11.9±0.8*^┼§^ |
| Paw delta | 1.0±0.1 | 2.3±0.3 | 3.6±0.4 | 12.6±1.1*^┼^ | 6.4±1.4*^┼§^ |
| Values are shown as mean ± SEM (mm Hg). All pressures are in mm Hg. Data are derived from 8 animals. * denotes p< 0.05 for STD vs. (ACD, ACD ITD, ACD ITPR). ^┼^ denotes p<0.05 for ACD vs. (ACD ITD, ACD ITPR). ^§^ denotes p<0.05 for ACD-ITD vs. ACD-ITPR. CPR, cardiopulmonary resuscitation; STD, standard; ACD, active compression decompression; ITD, impedance threshold device; ITPR, intrathoracic pressure regulator; Ppl, intrapleural pressure; Paw, Airway pressure; max, maximum (compression); min, minimum (decompression). | | | | | |
