# Supplementary material for: Effect of regulating airway pressure on intrathoracic pressure and vital organ perfusion pressure during cardiopulmonary resuscitation: a non-randomized interventional cross-over study
Source: Scand J Trauma Resusc Emerg Med. 2015 Oct 28;23:83. doi: 10.1186/s13049-015-0164-5 (PMC4625943; doi:10.1186/s13049-015-0164-5)
Supplement: Additional file 2: Figure S1. — Scatterplot depicting relationships between Ppl mean and Paw mean (A), Ppl decompression and Paw decompression (B), Ppl decompression and CPP (C) and, Ppl decompression and CePP (D). Ppl, Intrapleural pressure; Paw, airway pressure; CPP, coronary perfusion pressure; CePP, cerebral perfusion pressure. (DOCX 50 kb) [file 13049_2015_164_MOESM2_ESM.docx]

Supplemental Figure 1. Scatterplot depicting relationships between Ppl mean and Paw mean (A), Ppl decompression and Paw decompression (B), Ppl decompression and CPP (C) and, Ppl decompression and CePP (D). Ppl, Intrapleural pressure; Paw, airway pressure; CPP, coronary perfusion pressure; CePP, cerebral perfusion pressure.
